# 10th anniversary of allergy, asthma & clinical immunology

**DOI:** 10.1186/1710-1492-10-51

**Published:** 2014-10-22

**Authors:** Richard John Warrington, Paul Kevin Keith

**Affiliations:** Departments of Medicine & Immunology, University of Manitoba, GC310 820 Sherbrook Street, Winnipeg, MB R3A 1R9 Canada; Department of Medicine, McMaster University, Health Sciences Centre 3V47, 1280 Main Street West, Hamilton, ON L8S 4K1 Canada

The Canadian Society of Allergy & Clinical Immunology, founded in 1945, published its first Journal in 1996, as the Canadian Journal of Allergy & Clinical Immunology, under the Editorship of Dr Gordon Sussman. It was a hard copy journal containing mostly solicited manuscripts and its circulation was limited primarily to Society members. In 2004, *Allergy, Asthma & Clinical Immunology* (*AACI*) was founded, and was published by BC Decker from that year until 2009. In 2009, a decision was made to change to an electronic format and the Journal joined BioMed Central.

It is interesting to note that a number of our most accessed papers were originally submitted to the hard copy version of the Journal produced by BC Decker (see Table 1). These articles became generally available because of Open Access with BioMed Central. This demonstrates the importance of the Open Access system for the dissemination of medical and scientific information, for at its peak, the hard copy of *AACI* never had more than a few hundred subscribers, compared to the 20,000 to 35,000 accesses to the articles when they transferred to an electronic format.

**Table 1:** **Highly accessed articles (retrieved July 2014)**

Title		Year published	Article type	Online accesses
1.	Canadian clinical practice guidelines for acute and chronic rhinosinusitis.	2011	Guideline	34638
2.	Urticaria and infections	2009	Review	27945
3.	Adrenal suppression: A practical guide to the screening and management of under-recognized complication of inhaled corticosteroid therapy.	2011	Review	24240
4.	Urticaria and angioedema.	2011	Review	22028
5.	International consensus algorithm for the diagnosis, therapy and management of hereditary angioedema	2010	Guideline	21480
6.	The role of Probiotics in allergic diseases.	2009	Review	20857
7.	Allergic rhinitis.	2011	Review	19923
8.	Hodgkin’s lymphoma presenting with markedly elevated IgE: a case report	2009	Case Report	19033
9.	A practical guide to the monitoring and management of the complications of systemic corticosteroid therapy	2013	Review	18269
10.	Acquired angioedema	2010	Review	16277

Since joining BioMed Central, under the Editorships of Drs. Warrington, Kim & Watson, *Allergy Asthma & Clinical Immunology* has been indexed on PubMed, PubMed Central, Science Citation Index Expanded, Current Contents, & Scopus. *AACI* has been tracked by Thompson-Reuters for an Impact Factor since early 2014, and had an unofficial 2013 impact factor of 1.57. The Scopus Journal Rank (SJR) has increased progressively since 2009 from 0.1 to 0.5-0.6 in 2013 and the Source Normalized Impact per paper (SNIP) has increased from <0.1 to >0.8.

The number of manuscript publications has also increased progressively since 2010 from 33 in that year to 50 in 2013, the number in 2014 was at 40 as of July 2014.

Regarding the Country of origin of the manuscripts published in the Journal (Table 2), the majority are from Canada, perhaps not surprisingly, with USA the second highest contributing country. It is rather striking how few submissions and publications are from the UK. This area probably needs more attention. Perhaps this reflects the small number of Allergy & Clinical Immunology training programs in the UK and the fact they have their own journal, *Clinical & Experimental Allergy*. The UK is still third on the list of page visits.

**Table 2:** **Country of origin of publication since 2009**

Country	Numbers of publications
Canada	65
USA	27
Germany	11
Japan	8
UK	5
Italy	4
France	4
Others	44

Citations have also increased since joining BioMed Central, from <10 in 2009 to 240 in 2013, an indication of the growing interest in articles published in the Journal.

In terms of citations, it appears that guidelines are more frequently cited than other manuscripts, although we do not have information on the citation of our Primer on Allergy & Immunology which was published as a supplement. Articles in that supplement are among our most highly accessed.

When we compare highly accessed articles all time with those accessed in the last month (Table 3), there is a significant change in the article types, with a much higher proportion of manuscripts that are research articles being highly accessed, rather than reviews or guidelines, which is probably to be expected.

**Table 3:** **Highly accessed articles in last month**

Title		Year published	Article type	Online accesses last 30 days
1.	Auto-injector needle length may be inadequate to deliver epinephrine intramuscularly in women with confirmed food allergy.	2014	Research	3117
2.	Do epinephrine auto-injectors have an unsuitable needle length for young children?	2014	Meeting abstract	3353
3.	A practical guide to the monitoring and management of the complications of systemic corticosteroid therapy.	2013	Research	2246
4.	Children under 15 kg with food allergy may be at risk of having epinephrine auto-injectors administered into bone.	2014	Research	1516
5.	Adrenal suppression: A practical guide to the screening and management of this under-recognized complication of inhaled corticosteroid therapy.	2011	Guideline	1551
6.	Effect of ketotifen premedication on adverse reactions during peanut oral immunotherapy.	2014	Research	1253
7.	The potential mechanistic link between allergy and obesity development and infant formula feeding.	2014	Review	1212
8.	Urticaria and angioedema.	2011	Review	1202
9.	Allergic rhinitis.	2011	Review	1102
10.	Peanut Allergy: An Overview.	2008	Review	1047

*AACI* has also been successful in its publication of supplements. In addition to the yearly supplement containing abstracts from the Annual Meeting of the Canadian Society of Allergy & Clinical Immunology, we also publish abstracts from the Allergen NCE Inc. Annual Meeting, recently held in conjunction with the CSACI Meeting. This extremely valuable collaboration enhances and expands the basic science interest in the Annual Meeting and facilitates interactions between basic scientists, clinician scientists and clinicians.

A second successful contribution by *AACI* has been in the form of an educational supplement providing review articles on Allergy & Clinical Immunology topics suitable for Medical School teaching and the education of non-specialist physicians. The articles in this supplement have also been among the most accessed publications in *AACI*. This emphasizes the strong educational value of the open access system particularly as the cost of textbooks in Allergy & Immunology increases progressively.

A third successful source of information in *AACI* is in the form of guidelines for the treatment and management of allergic and immunologic disease. Some frequently accessed examples of these guidelines are provided in Table 1.

Reviewing data from Google Analytics, the number of page views per month has increased progressively to 34,660 in the month of July. Of these, 15,112 were unique visitors, while new visitors are typically about 20% of all visits. Of these, the majority are from the United States, followed by Canada, India and the United Kingdom. The number of new visitors is encouraging with regard to the increasing visibility of the Journal and interest in its content. Approximately 12,000 sessions originate from Google searches, compared to 189 from Bing and 143 from Yahoo. Two thousand one hundred and twenty visits were direct and 668 were from BioMed Central. The latter had the greatest number of page session, at 5.15, compared to 1.66 from Google and 1.59 from PubMed. Direct visits, most likely from return visitors, viewed 2.48 pages per session. These data are interesting in terms of the methods of access to the journal, with a greater number of visitors from search engines, who view only a limited number of pages, compared to visitors who come direct to the Journal or through BioMed Central.

In this, our 10^th^ year of publication, we would like to thank the Co-editors of *AACI*, the Editorial Board, who have worked hard to make the Journal a success, and the many reviewers who have contributed their time and expertise.

In addition, we thank the Canadian Society of Allergy & Clinical Immunology, the Executive and Board and Management team without whose support, both financial and by their contributions to the Journal; we would not have achieved this success.

Finally, we must thank the many staff at BioMed Central that have contributed to our success. A special thanks to Lisa Hussey and Holly Young, who have guided the Journal through the last several years successfully.

When *AACI* first began publishing in 2004, our goal was to publish high quality research and review manuscripts, with the aim of eventually achieving indexing in PubMed, Current Contents etc. Our manuscripts were then by invitation only. Since joining BioMed Central, we have achieved the aim of indexing, including now Science Citation Index Expanded, and are awaiting our first official Impact Factor from Thompson Reuters. What we wish to achieve in the next 10 years is a steady increase in the number and quality of submissions, so that we can enhance the international reputation of our journal and Society. This can be achieved by taking advantage of the tremendous advances in our understanding of allergic and immunologic diseases and the new treatments that have become available. This knowledge can be widely disseminated by the important medium of Open Access.

Richard Warrington, Editor-in-Chief, Allergy Asthma & Clinical Immunology

Paul Keith, President, Canadian Society of Allergy & Clinical Immunology

